# Natural Th1 cells: escape from neglect

**DOI:** 10.18632/oncotarget.5489

**Published:** 2015-08-11

**Authors:** Seong Hoe Park

**Affiliations:** Transplantation Research Institute, Medical Research Center, Seoul National University College of Medicine, Seoul, Korea

**Keywords:** natural Th1 cells, innate T cells, eomes, T-T interaction, Immunology and Microbiology Section, Immune response, Immunity

A novel idea that there is an alternative pathway for intrathymic generation of mature CD4^+^ T cells came from the observation that immature thymocytes were also able to express MHC class II on their cell surfaces during fetal thymic development [1]. This idea was subsequently confirmed both by *in vitro* reaggregate thymic organ culture (RTOC) [2] and *in vivo* plck-CIITA^tg^ mouse system [3, 4] where MHC class II molecules are designed to be expressed only on T cells. These CD4 T cells selected via T-T interaction has been, therefore, referred to as T-T CD4^+^ T cells. Consequently, this novel pathway creates very intriguing and important innate T cell subsets; PLZF^+^ T-T CD4 T cells [5], Eomes^+^ innate CD8 T cells [6] and, last but not at the least, Eomes^+^ natural Th1 cells [7].

Of these three different types of innate T cells, PLZF^+^ T-T CD4 T cells are developed via T-T interaction in thymus, whereas both nTh1 and Eomes^+^ innate CD8 T cells are generated via bystander effect in the presence of Promyelocytic leukemia zinc-finger protein (PLZF)-positive T-T CD4 T cells [6, 7]. PLZF^+^ T-T CD4 T cells shared several functional characteristics with iNKT cells such as rapid production of both IL-4 and IFN-γ upon TCR stimulation, and the unique dependence on the SLAM-SAP signaling pathway. However, PLZF^+^ T-T CD4 T cells and iNKT cells are really rather different in one key aspect; that PLZF^+^ T-T CD4 T cells have extreme TCR diversity as was in conventional CD4 T cells [5].

IL-4 produced by PLZF^+^ T-T CD4 T or NKT cells induces the generation of Eomes^+^ innate CD8 and nTh1 cells [6, 7]. Eomes^+^ innate CD8 and nTh1 cells are in fact extraordinarily susceptible to IL-4 and have much higher number of IL-4 receptor in their precursor cells which were produced right after positive selection. Eomes^+^ innate CD8 T cells produced large amounts of IFN-γ and TNF-α within a few hours upon TCR stimulation. The functional significance of this cell population was further highlighted by their ability to rapidly clear viremia during chronic lymphocytic choriomeningitis virus (LCMV) infection (unpublished observations).

**Figure 1:**
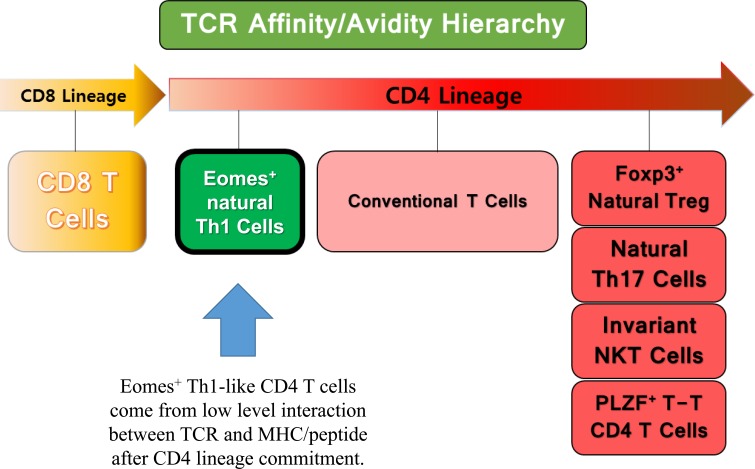
A hierarchy of TCR affinity/avidity of developing T cells PLZF^+^ T-T CD4 T cells, invariant NKT cells and natural Th17 cells are developed with high affinity/avidity interaction, whereas converse is true for this Eomes^+^ nTh1 cells.

Remarkable specialization that nTh1 cells have is that a low affinity/avidity interaction selects this type of T cells during thymic positive selection. A series of events underlies the generation of Eomes^+^ nTh1 cells; low affinity/avidity interaction between the TCR and MHC peptide, increased susceptibility to common γ-chain (γc) cytokines (in this case, IL-4), concomitant expression of IL-4Rα and Eomes, and, finally, dramatic upregulation of anti-apoptotic protein Bcl-2 for their survival. This mechanism appears to work contrary to other types of innate cells, such as iNKT cells, nTh17 cells, and PLZF^+^ T-T CD4 T cells, which receive high levels of signals from the interaction between the MHC/peptide and the appropriate TCR (Figure 1). All of these events dramatically illustrate the fact that there are inverse relationship between signal strength received by T cells and γc cytokine susceptibility. T-T interaction and subsequent generation of PLZF^+^ T-T CD4 T cells seem to exist in wide range of mammalians, and generation of three types of innate T cells via T-T interaction takes place in human system as well [5–7]. Especially, we discovered that both Eomes^+^ CD4 and CD8 T cells exist not only in cord blood cells but also in adult human peripheral blood mononuclear cells (unpublished observations).

Based on a variety of consideration, we propose that the biological relevance of T-T interaction is the generation of early effector T cells against diverse foreign pathogens, particularly viruses. From an evolutionary point of view, the limited TCR diversity of mouse innate T-cell subsets (e.g. iNKT cells, H2-M3-specific T cells and MR1-specific T cells) more serves to defend against bacteria, whereas nTh1 cells and Eomes^+^ innate CD8 T cells with their extremely diverse TCR repertoire would be more effective in defending against viral infections. Of note, nTh1 cells express stem-like memory T cell markers (CD45R0^−^, CCR7^+^, CD45RA^+^, CD62L^+^, CD27^+^, CD28^+^, IL-2Rβ^+^ and CXCR3^+^) particularly in human. Considering the fact that human thymus starts to involute relatively early in their life, stem-like peripheral Eomes^+^ T cells with their potential homeostatic proliferation might maintain a competent anti-viral defense even long after complete thymic involution. Since nTh1 cells have gone through low affinity/avidity interaction during entire thymocyte maturation pathway, they have relatively low chances to see their self-antigens in the periphery to initiate autoimmunity.

In summary, T-T interaction is the real basis for the development of three different types (PLZF^+^ CD4 T, and Eomes^+^ CD4 and CD8 T cells) of innate T cells both in mouse and human immune system. We again would like to emphasize the possibility that Eomes^+^ nTh1 cells with innate-like properties but with diverse TCR repertoire might play a critical role to defend against extremely diverse exogenous pathogens, particularly viruses.
